# The association between hemoglobin concentration and clinical outcomes after aneurysmal subarachnoid hemorrhage: Insights from the LongTEAM registry

**DOI:** 10.1111/cns.14506

**Published:** 2023-10-17

**Authors:** Fa Lin, Changyu Lu, Runting Li, Yu Chen, Heze Han, Yuanli Zhao, Xiaolin Chen, Jizong Zhao

**Affiliations:** ^1^ Department of Neurosurgery, Beijing Tiantan Hospital Capital Medical University Beijing China; ^2^ China National Clinical Research Center for Neurological Diseases Beijing China; ^3^ Department of Neurosurgery Peking University International Hospital Beijing China

**Keywords:** aneurysmal subarachnoid hemorrhage, deep vein thrombosis, hemoglobin, outcome, treatment

## Abstract

**Objective:**

The aim of this study is to explore whether preoperative hemoglobin levels could serve as a prospective biomarker for early brain injury in patients with aneurysmal subarachnoid hemorrhage (aSAH). This investigation seeks to discern its association with postoperative complications and unfavorable clinical outcomes.

**Methods:**

We conducted a comprehensive analysis of data derived from the LongTeam registry, including patients with aSAH diagnosed between January 2015 and September 2021. These patients were stratified into three distinct groups based on their hemoglobin levels: anemic, standard, and elevated HGB. We employed logistic models featuring spline transformations to assess the relationship between HGB levels and in‐hospital complications. Furthermore, a multivariate Cox proportional hazard model was employed to estimate the impact of elevated hemoglobin levels on the hazard function, which was elucidated through Kaplan–Meier curves.

**Results:**

Our study comprised a total of 988 patients, among whom 115 (11.6%) presented preoperative anemia, and 63 (6.4%) exhibited elevated preoperative HGB levels. Following adjustments for potential confounding factors, no significant disparity in risk was evident between anemic patients and those with standard HGB levels. However, individuals with elevated HGB levels displayed a heightened incidence and an increased risk of developing deep vein thrombosis (DVT, odds ratio [OR] = 2.39, 95% confidence interval [CI] = 1.16–4.91, *p* = 0.018; hazard ratio [HR] = 2.05, 95% CI 1.08–3.92, *p* = 0.015). Aberrant HGB concentrations did not demonstrate an association with other clinical outcomes.

**Conclusion:**

Our findings emphasize that abnormal HGB levels show no association with adverse outcomes at the 90 days mark after accounting for clinical confounding factors in patients with aSAH. Simultaneously, the study illuminates the potential of HGB as an early indicator for identifying patients at a heightened risk of developing DVT.

## Graphical abstract

Our findings demonstrate a significant correlation between preoperative high HGB concentrations and the occurrence of DVT following aSAH. This highlights the potential of HGB as an early indicator for identifying patients at high risk of developing DVT.

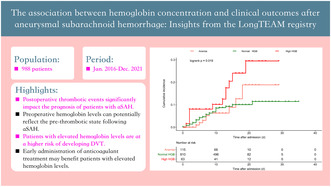

## 1 INTRODUCTION

Aneurysmal subarachnoid hemorrhage (aSAH) is a life‐threatening condition characterized by high morbidity and mortality.^1^, ^2^ Currently, researchers acknowledge two pivotal phases profoundly affecting the prognosis of aSAH patients: the early brain injury (EBI) phase and the subsequent phase of delayed complications.^3^ These two periods are intricately intertwined, with EBI capable of exacerbating the onset of delayed complications. EBI encompasses the pathophysiological mechanisms unfolding within 72 h post‐aneurysm rupture, excluding iatrogenic injuries. The pathophysiological alterations associated with EBI not only impact the brain but also give rise to systemic complications, precipitating adverse patient outcomes.^4^ Hence, identifying significant indicators of EBI and its downstream complications is of paramount importance for predicting patient prognosis.

Numerous investigations have underscored the significance of preoperative laboratory assessments in aSAH patients, positing that specific preoperative laboratory parameters can elucidate the pathophysiological trajectory entailed in EBI. Consequently, these metrics hold considerable value in forecasting complications and clinical consequences.^5^, ^6^, ^7^, ^8^, ^9^ Hemoglobin (HGB), an intricate protein responsible for oxygen conveyance within erythrocytes, stands as an efficacious indicator of anemia severity.^10^ While previous studies have mainly primarily focused on ischemic stroke rather than hemorrhagic stroke, they have corroborated that aberrant preoperative HGB concentrations can heighten the risk of stroke recurrence, vascular events, and unfavorable outcomes.^11^, ^12^, ^13^ Nevertheless, the scrutiny of preoperative HGB concentrations in aSAH patients and their relevance in predicting clinical outcomes post‐aSAH remains insufficient.^14^, ^15^

This study endeavors to appraise the association between preoperative HGB concentration and clinical outcomes subsequent to aSAH, encompassing 10 in‐hospital complications and 90 days functional outcomes. These findings will furnish healthcare practitioners with the means to identify patients at high risk of adverse outcomes, enabling the implementation of more efficacious preventive strategies.

## 2 METHODS

### 2.1 Study design

Patient data were collected from the LongTEAM registry study (Registration No. NCT 04785976), a comprehensive, single‐center, observational cohort conducted at Beijing Tiantan Hospital in China. ^16^, ^17^ ^)^ The data collection period extended from January 2015 to September 2021. Diagnosis of aSAH was established through various imaging techniques, including computed tomography, computed tomography angiography, digital subtraction angiography, or lumbar puncture. Inclusion criteria encompassed the following: (1) age ≥ 18 years; (2) emergent admission; (3) manifestation of a single aneurysm; (4) receipt of surgical clipping or endovascular coiling; (5) a time interval between rupture and admission, and from admission to treatment, both within a 72 h window. On the contrary, exclusion criteria encompassed patients lacking baseline HGB levels, those with sickle cell disease, cancer, previous SAH, or physical disability resulting from antecedent illnesses. Furthermore, patients lost to follow‐up within 90 days post‐discharge were also excluded from the study.

This study duly obtained approval from the ethics committee of Beijing Tiantan Hospital (KY 2021–008‐01). Thoroughly informed consent for clinical analysis was acquired from all individual participants or their authorized representatives. The analysis strictly adhered to the principles of the Declaration of Helsinki and local ethics policies. Patient management adhered to well‐established guidelines.^18^

### 2.2 Data collection

Data collection involved trained neurosurgeons who gathered demographic information, medical histories, lifestyle‐related risk factors, details regarding the location and size of ruptured aneurysms, symptoms experienced after onset, clinical status (including World Federation of Neurological Societies [WFNS] grade, modified Fisher scale [mFS], Graeb score, Subarachnoid Hemorrhage Early Brain Edema Score [SEBES], and acute hydrocephalus), and the employed treatment modalities. Concurrently, laboratory examinations, including HGB concentration measurement, were routinely performed within 24 h of admission.

Furthermore, comprehensive data were amassed concerning 10 in‐hospital complications: namely cardiac events, delayed cerebral ischemia (DCI), intracranial infection, stress ulcer bleeding, urinary tract infection, hypoproteinemia, pneumonia, muscular calf vein thrombosis (MCVT), deep vein thrombosis (DVT), and lipid metabolism disorder. Detailed diagnostic criteria are accessible in Table S1.

### 2.3 Outcome assessment

Neurosurgeons conducted follow‐up with patients either through telephone consultations or outpatient appointments. The primary outcome was the modified Rankin Scale (mRS) at 90 days post‐discharge. The unfavorable outcome was defined as an mRS score ≥3. The secondary outcomes were the occurrence of in‐hospital complications.

### 2.4 Statistical analysis

We categorized the patients into three groups based on World Health Organization (WHO) criteria and established research: anemic (hemoglobin <120 g/L for women, hemoglobin <130 g/L for men), standard hemoglobin (hemoglobin 120–155 g/L for women, hemoglobin 130–170 g/L for men), and elevated hemoglobin (hemoglobin >155 g/L for women, hemoglobin >170 g/L for men).^19^, ^20^ Continuous variables were presented as mean ± standard deviation (SD) for normally distributed data and as median with interquartile range (IQR) for skewed distributions. Categorical variables were expressed as numerical values (percentages). Baseline characteristics among the three groups were compared using chi‐square test for categorical variables, ANOVA test for normal distributed continuous variables, and the nonparametric Kruskal‐Wallis test for skewed distributed continuous variables.

To evaluate the relationship between HGB levels and clinical outcomes, we employed a logistic regression model. HGB level were discretized into a categorical variable with three tiers: anemic, standard, and elevated. Clinical outcomes were evaluated using binary indicators to determine the occurrence of in‐hospital complications and whether the 90 days mRS score exceeded 3. We constructed three nested models for each binary outcome, incorporating various levels of adjustment:(1) Model 1 adjusted for age, gender, and treatment; (2) Model 2 further adjusted for medical history, including additional covariates such as hypertension, heart disease, current smoking, and current drinking; (3) Model 3 additionally considered WFNS grade, Graeb score, and SEBES score to address heterogeneity in patients' initial clinical status. With regard to the primary outcome, we incorporate DCI into Model 3. Adjusted odds ratio (OR) with 95% confidence intervals (CIs) were calculated, using the standard group as the reference. To visualize the nonlinear correlation between HGB and outcome variables, we developed a logistic model that integrated HGB as a restricted cubic spline with three knots.

The Kaplan–Meier method was utilized to estimate the cumulative incidence of DVT over the follow‐up period, with the log‐rank test employed to assess risk differences among the three HGB groups. A multivariate Cox proportional hazard (PH) regression model was fitted to estimate the impact of HGB on the hazard function of developing in‐hospital DVT. Hazard ratios (HR) with 95%CIs were calculated, with the standard group serving as the reference.

Furthermore, we conducted subgroup analysis, stratifying by age (>55 and ≤ 55 years), gender (male and female), hypertension (yes and no), WFNS grade (1–3 and 4–5), and treatment modality (surgical clipping and endovascular coiling). This analysis aimed to identify any significant interactions between these factors and HGB concentrations when assessing in‐hospital DVT.

The analysis was performed using the following software: SPSS Statistics 26.0 (IBM), R software (Version 4.1.2), and GraphPad PRISM 8.3.0. (GraphPad Software Inc,). The significance level was set at *p* < 0.05.

## 3 RESULTS

A total of 988 patients diagnosed with aSAH were included in this study. Within this cohort, the mean age was 54.8 years, with females constituting 59.0%. Upon admission, 115 patients (11.6%) presented with anemia, while 63 patients (6.4%) displayed elevated HGB levels. Upon comparative analysis of the three groups, it was discerned that patients with elevated HGB levels had a higher prevalence of medical history, including conditions such as hypertension (71.4%) and heart disease (14.3%). Moreover, patients with elevated HGB levels exhibited a larger proportion of current smokers (33.3%), WFNS grade 4–5 (36.5%), Graeb score 5–12 (22.2%), and SEBES score 3–4 (71.4%). Conversely, individuals with preoperative anemia demonstrated a higher percentage of female patients (73.9%), whereas those with standard HGB levels had a higher incidence of current alcohol consumption (22.0%). Refer to Table 1 for a comprehensive overview of the patients' baseline characteristics according to HGB concentration groups.

**TABLE 1:** Baseline information.

Characteristics	Total	Hemoglobin groups			*p*
		Anemia	Normal	High	
No. of patients	988	115	810	63	
Age, years, mean ± SD	54.8 ± 11.0	56.0 ± 12.2	54.7 ± 11.0	54.5 ± 9.9	0.233
Female, *n* (%)	583 (59.0)	85 (73.9)	459 (56.7)	39 (61.9)	0.002
Hypertension, *n* (%)	581 (58.8)	56 (48.7)	480 (59.3)	45 (71.4)	0.011
Hyperlipidemia, *n* (%)	78 (7.9)	5 (4.3)	67 (8.3)	6 (9.5)	0.305
Diabetes mellitus, *n* (%)	92 (9.3)	12 (10.4)	70 (8.6)	10 (15.9)	0.149
Heart disease, *n* (%)	74 (7.5)	12 (10.4)	53 (6.5)	9 (14.3)	0.035
Current smoking, *n* (%)	263 (26.6)	18 (15.7)	224 (27.7)	21 (33.3)	0.011
Current drinking, *n* (%)	198 (20.0)	9 (7.8)	178 (22.0)	11 (17.5)	0.002
Antiplatelet agents, *n* (%)	44 (4.5)	3 (2.6)	38 (4.7)	3 (4.8)	0.594
Posterior circulation, *n* (%)	106 (10.7)	14 (12.2)	86 (10.6)	6 (9.5)	0.837
Maximum diameter of aneurysm, mean ± SD	6.6 ± 5.4	6.6 ± 7.1	6.5 ± 5.2	6.7 ± 4.8	0.456
Early seizures, *n* (%)	54 (5.5)	5 (4.3)	46 (5.7)	3 (4.8)	0.815
Early loss of consciousness, *n* (%)	304 (30.8)	35 (30.4)	245 (30.2)	24 (38.1)	0.428
WFNS grade 4–5, *n* (%)	222 (22.5)	20 (17.4)	179 (22.1)	23 (36.5)	0.012
mFS grade 3–4, *n* (%)	718 (72.7)	80 (69.6)	594 (73.3)	44 (69.8)	0.609
Graeb score 5–12, *n* (%)	84 (8.5)	5 (4.3)	65 (8.0)	14 (22.2)	<0.001
SEBES score 3–4, *n* (%)	491 (49.7)	50 (43.5)	396 (48.9)	45 (71.4)	0.001
Acute hydrocephalus, *n* (%)	387 (39.2)	43 (37.4)	314 (38.8)	30 (47.6)	0.351
Treatment modality					0.916
Surgical clipping, *n* (%)	507 (51.3)	57 (49.6)	418 (51.6)	32 (50.8)	
Endovascular coiling, *n* (%)	481 (48.7)	58 (50.4)	392 (48.4)	31 (49.2)	

Abbreviations: mFS, modified Fisher; SEBES, subarachnoid hemorrhage early brain edema score; WFNS, world federation of neurological societies.

^a^ Unit of measurement: mm.

### 3.1 Hemoglobin concentration and clinical outcomes

We identified 181 (18.3%) patients with unfavorable outcomes at 90 days. There were 376 (38.1%) patients with cardiac events, 268 (27.1%) patients with DCI, 125 (12.7%) patients with intracranial infection, 209 (21.2%) patients with stress ulcer bleeding, 28 (2.8%) patients with urinary tract infection, 345 (34.9%) patients with hypoproteinemia, 309 (31.3%) patients with pneumonia, 252 (25.5%) patients with MCVT, 85 (8.6%) patients with DVT, 245 (24.8%) patients with lipid metabolic disorder. Table 2 presents the adjusted odds ratio (aOR) for HGB in relation to clinical outcomes, along with the corresponding 95%CIs. In model 1, patients with elevated HGB had increased incidences of stress ulcer bleeding (OR 1.80, 95%CI 1.02–3.19, *p* = 0.043), pneumonia (OR 2.38, 95%CI 1.41–4.04, *p* < 0.001), DVT (OR 2.90, 95%CI 1.45–5.78, *p* = 0.002), and unfavorable outcomes at 90 days (OR 2.21, 95%CI 1.22–4.00, *p* = 0.009), compared to patients with standard HGB. In model 2, patients with elevated HGB had higher incidences of pneumonia (OR 2.14, 95%CI 1.25–3.66, *p* = 0.006), DVT (OR 2.63, 95%CI 1.31–5.31, *p* = 0.007), and unfavorable outcomes at 90 days (OR 2.13, 95%CI 1.17–3.89, *p* = 0.013), compared to patients with standard HGB. In the fully adjusted model (model 3), patients with elevated HGB were only associated with a higher incidence of DVT (OR 2.39, 95%CI 1.16–4.91, *p* = 0.018), compared to patients with standard HGB. However, the primary endpoint did not demonstrate statistical significance.

**TABLE 2:** Adjusted odds ratios for clinical outcomes according to the hemoglobin groups.

Variables	HGB	No.	Model 1			Model 2		Model 3	
			Events, *n* (%)	Adjusted OR (95% CI)	*p*	Adjusted OR (95% CI)	*p*	Adjusted OR (95% CI)	*p*
90 days unfavorable outcome	Anemic	115	16 (13.9)	0.63 (0.35–1.14)	0.127	0.65 (0.36–1.18)	0.160	0.78 (0.40–1.51)	0.454
	Standard	810	146 (18.0)	–	–	–	–	–	–
	Elevated	63	19 (30.2)	2.21 (1.22–4.00)	**0.009**	2.13 (1.17–3.89)	**0.013**	1.60 (0.78–3.32)	0.203
Cardiac event	Anemic	115	40 (34.8)	0.84 (0.55–1.29)	0.431	0.91 (0.59–1.40)	0.658	0.94 (0.61–1.45)	0.780
	Standard	810	308 (38.0)	–	–	–	–	–	–
	Elevated	63	28 (44.4)	1.34 (0.79–2.28)	0.273	1.25 (0.72–2.15)	0.426	1.11 (0.64–1.94)	0.712
DCI	Anemic	115	24 (20.9)	0.65 (0.40–1.06)	0.082	0.67 (0.41–1.09)	0.108	0.71 (0.43–1.16)	0.169
	Standard	810	229 (28.3)	–	–	–	–	–	–
	Elevated	63	15 (23.8)	0.80 (0.44–1.48)	0.481	0.79 (0.43–1.47)	0.462	0.66 (0.35–1.25)	0.204
Intracranial infection	Anemic	115	16 (13.9)	1.25 (0.68–2.31)	0.472	1.25 (0.68–2.32)	0.476	1.31 (0.70–2.43)	0.397
	Standard	810	102 (12.6)	–	–	–	–	–	–
	Elevated	63	7 (11.1)	0.88 (0.38–2.08)	0.774	0.94 (0.40–2.23)	0.887	0.84 (0.35–2.03)	0.696
Stress ulcer bleeding	Anemic	115	30 (26.1)	1.46 (0.92–2.31)	0.108	1.50 (0.94–2.40)	0.088	1.60 (0.99–2.57)	0.052
	Standard	810	160 (19.8)	–	–	–	–	–	–
	Elevated	63	19 (30.2)	1.80 (1.02–3.19)	**0.043**	1.63 (0.91–2.93)	0.100	1.43 (0.78–2.61)	0.245
Urinary tract infection	Anemic	115	4 (3.5)	1.20 (0.40–3.60)	0.739	1.13 (0.37–3.45)	0.827	1.21 (0.40–3.72)	0.736
	Standard	810	22 (2.7)	–	–	–	–	–	–
	Elevated	63	2 (3.2)	1.15 (0.26–5.03)	0.857	1.00 (0.22–4.51)	0.996	0.83 (0.18–3.80)	0.812
Hypoproteinemia	Anemic	115	36 (31.1)	0.81 (0.51–1.27)	0.351	0.80 (0.51–1.25)	0.320	0.87 (0.55–1.37)	0.544
	Standard	810	284 (35.1)	–	–	–	–	–	–
	Elevated	63	25 (39.7)	1.30 (0.75–2.26)	0.353	1.30 (0.74–2.26)	0.364	1.00 (0.56–1.80)	0.996
Pneumonia	Anemic	115	34 (29.6)	0.97 (0.63–1.51)	0.897	0.98 (0.63–1.54)	0.938	1.14 (0.71–1.84)	0.578
	Standard	810	244 (30.1)	–	–	–	–	–	–
	Elevated	63	31 (49.2)	2.38 (1.41–4.04)	**0.001**	2.14 (1.25–3.66)	**0.006**	1.59 (0.88–2.87)	0.126
MCVT	Anemic	115	29 (25.2)	0.88 (0.55–1.41)	0.592	0.89 (0.56–1.43)	0.638	0.96 (0.59–1.54)	0.853
	Standard	810	203 (25.1)	–	–	–	–	–	–
	Elevated	63	20 (31.7)	1.46 (0.82–2.60)	0.195	1.46 (0.82–2.60)	0.203	1.16 (0.63–2.12)	0.638
DVT	Anemic	115	9 (7.8)	0.91 (0.44–1.90)	0.798	0.93 (0.44–1.97)	0.857	0.98 (0.46–2.07)	0.951
	Standard	810	64 (7.9)	–	–	–	–	–	–
	Elevated	63	12 (19.0)	2.90 (1.45–5.78)	**0.002**	2.63 (1.31–5.31)	**0.007**	2.39 (1.16–4.91)	**0.018**
Lipid metabolism disorder	Anemic	115	20 (17.4)	0.63 (0.38–1.05)	0.075	0.70 (0.42–1.18)	0.184	0.67 (0.40–1.14)	0.142
	Standard	810	205 (25.3)	–	–	–	–	–	–
	Elevated	63	20 (31.7)	1.39 (0.79–2.43)	0.250	1.38 (0.78–2.45)	0.272	1.51 (0.84–2.71)	0.166

*Note*: Model 1: adjusted for age + sex + treatment modality.

Model 2: adjusted for model 1 + hypertension + heart disease + current smoking + current drinking.

Model 3: adjusted for model 1 + model 2 + WFNS 4–5 + Graeb 5–12 + SEBES 3–4.

For primary outcome, Model 3: adjusted for model 1 + model 2 + WFNS 4–5 + Graeb 5–12 + SEBES 3–4 + DCI.

Abbreviations: DCI, delayed cerebral ischemia; DVT, deep vein thrombosis; MCVT, muscular calf vein thrombosis.

**FIGURE 1:**
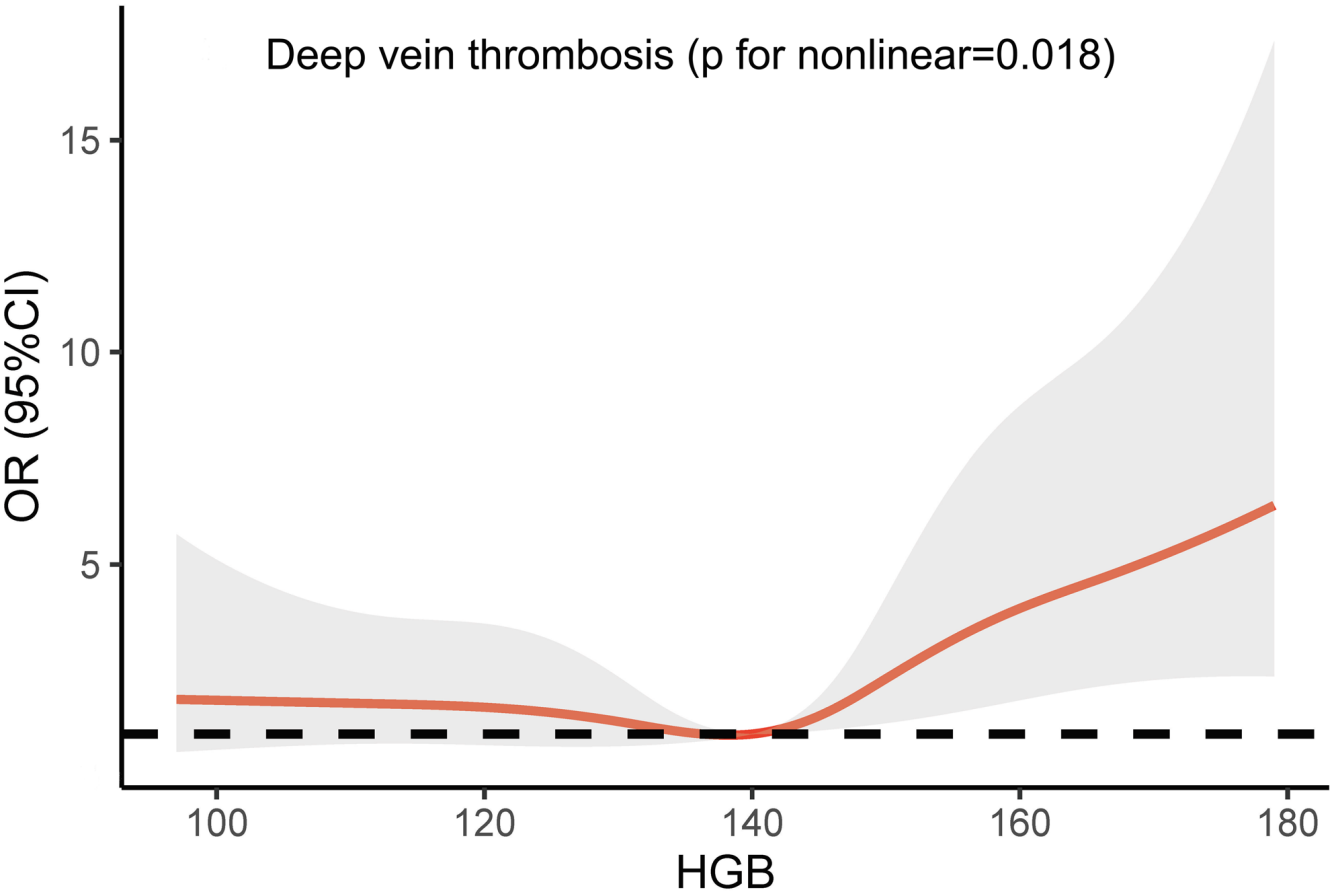
Adjusted odds ratios of DVT according to baseline HGB concentrations. The reference is the median of the baseline HGB concentration (140 g/L). Data were fitted using a logistic regression model of the restricted cubic spline with five knots (the 5th, 25th, 50th, 75th, and 95th percentiles) for baseline HGB concentration, adjusting for potential covariates (model 3).

Figure 1 illustrates the predicted OR for the onset of DVT as per the adjusted restricted cubic regression model, using HGB levels as the independent variable. The reference level (OR 1) was set at the median HGB level of 140 g/L. Initially, the curve exhibits a gentle incline but escalates notably for HGB levels surpassing the median, signifying that the divergence in risk between the anemic and standard group is inconsequential. Nevertheless, within the elevated HGB group, an elevation in HGB level translates into a substantial upswing in the adjusted risk.

**FIGURE 2:**
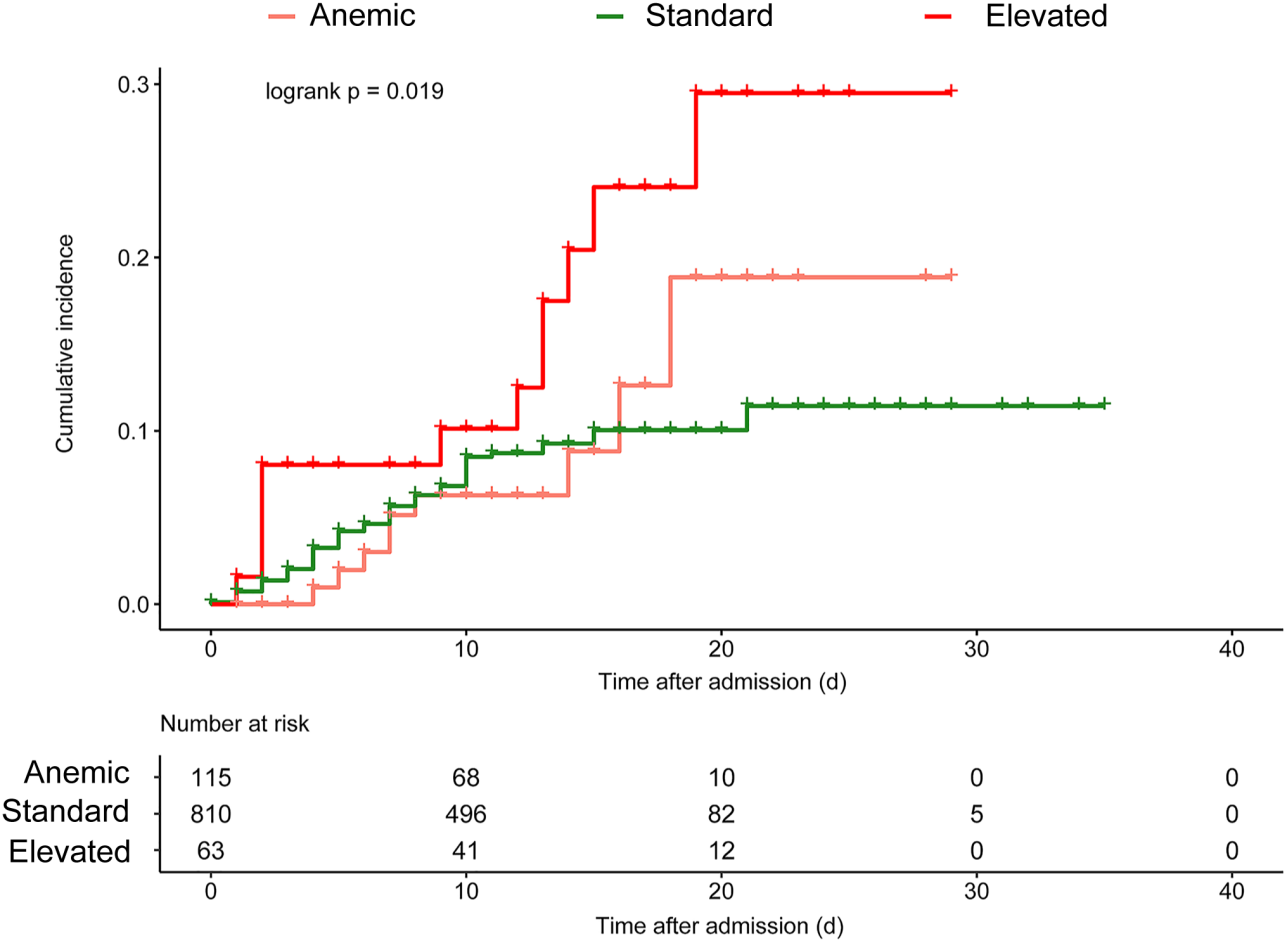
Kaplan–Meier curves for in‐hospital DVT.

The fully adjusted Cox PH model indicates a significantly augmented susceptibility to DVT within the elevated HGB group (HR = 2.05, 95%CI 1.08–3.92, *p* = 0.03), although no such elevation is observed in the anemic group (HR = 0.94, 95%CI 0.46–1.91, *p* = 0.86), compared to patients with standard HGB. Figure 2 illustrates the Kaplan–Meier estimations of cumulative incidence curves for DVT, stratified by HGB groups. The log‐rank test (*p* = 0.019) confirms noteworthy distinctions among the three groups, with pairwise comparisons revealing a significant difference solely between the elevated and standard groups (*p* = 0.016). These findings, as derived from the Cox model and Kaplan–Meier analysis, align harmoniously with the results of the logistic regression model.

### 3.2 Subgroup analysis

Table 3 presents the ORs pertaining to DVT based on age, gender, hypertension, WFNS grade, and treatment modality. Following the adjustment for all conceivable confounding factors as per model 3, no modifications in the likelihood of DVT were observed concerning age, gender, hypertension, WFNS grade, and treatment modality (all *p*‐values for interaction >0.05).

**TABLE 3:** Subgroup analysis of association between hemoglobin groups and in‐hospital deep vein thrombosis.

Subgroup	HGB groups	No. of patients	Events	Adjusted OR (95%CI)	*p* for interaction
Age					0.237
≤55	Anemic	59	2 (3.4)	0.97 (0.21–4.50)	
	Standard	427	21 (4.9)	–	
	Elevated	37	8 (21.6)	4.86 (1.78–13.22)	
>55	Anemic	56	7 (12.5)	1.10 (0.45–2.70)	
	Standard	383	43 (11.2)	–	
	Elevated	26	4 (15.4)	1.43 (0.44–4.60)	
Sex					0.423
Male	Anemic	30	2 (6.7)	1.11 (0.22–5.55)	
	Standard	351	21 (6.0)	–	
	Elevated	24	6 (25.0)	5.65 (1.70–18.73)	
Female	Anemic	85	7 (8.2)	0.94 (0.40–2.25)	
	Standard	458	43 (9.4)	–	
	Elevated	39	6 (15.4)	1.68 (0.63–4.49)	
Hypertension					0.594
Yes	Anemic	56	7 (12.5)	1.18 (0.49–2.83)	
	Standard	480	47 (9.8)	–	
	Elevated	45	11 (24.4)	2.94 (1.32–6.58)	
No	Anemic	59	2 (3.4)	0.60 (0.13–2.85)	
	Standard	330	17 (5.2)	–	
	Elevated	18	1 (5.6)	1.01 (0.11–9.39)	
WFNS grade					0.903
1–3	Anemic	95	6 (6.3)	0.83 (0.33–2.05)	
	Standard	631	42 (6.7)	–	
	Elevated	40	6 (15.0)	2.46 (0.92–6.56)	
4–5	Anemic	20	3 (15.0)	1.64 (0.39–6.93)	
	Standard	179	22 (12.3)	–	
	Elevated	23	6 (26.1)	2.64 (0.82–8.54)	
Treatment modality					0.150
Surgical clipping	Anemic	57	8 (14.0)	1.52 (0.64–3.62)	
	Standard	418	37 (8.9)	–	
	Elevated	32	9 (28.1)	3.57 (1.42–9.00)	
Endovascular coiling	Anemic	58	1 (1.7)	0.25 (0.03–1.89)	
	Standard	392	27 (6.9)	–	
	Elevated	31	3 (9.7)	1.09 (0.29–4.12)	

### 3.3 Time of developing DVT in different groups, and the relationship between DVT and length of hospital stay, number of complications, and total hospital charges

**FIGURE 3:**
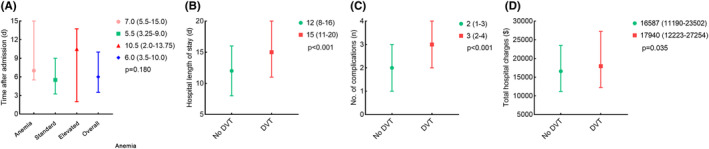
(A) The Timing of DVT formation. (B) The relationship between length of stays and DVT. (C) The relationship between the number of complications and DVT. (D) The relationship between total hospital charges and DVT. (1 US dollar = 7.05 renminbi [updated on September 21, 2022].

The median duration from admission to the onset of DVT exhibited no significant disparities among patients within the anemic, standard, and elevated HGB groups (7.0 [5.5–15.0] vs. 5.5 [3.3–9.0] vs. 10.5 [2.0–13.8], *p* = 0.180). Nevertheless, patients with DVT, in comparison to those without, incurred extended hospitalizations (15 [11–20] vs. 12 [8–16], *p* < 0.001), more complications (3 [2–4] vs. 2 [1–3], *p* < 0.001), and higher hospital expenditures (17,940 [12223–27,254] vs. 16,587 [11190–23,502], *p* = 0.035). Refer to Figure 3 for more detailed information.

## 4 DISCUSSION

In this expansive cohort study conducted in China, featuring a comprehensive analysis of in‐hospital complications and 90 days functional outcomes among aSAH patients, we have unveiled a notable association. It appears that individuals with elevated preoperative HGB, in contrast to those with standard preoperative HGB, exhibited an elevated susceptibility to in‐hospital DVT. However, such evidence was not observed among patients with anemia. Our findings emphasize the significance of this phenomenon, even after adjusting for the heterogeneity in age, gender, treatment modalities, medical history, and initial neurological assessments.

Previous investigations into HGB underscore the importance of managing post‐treatment anemia and advocate for a higher management target (110–120 g/L) for postoperative HGB in aSAH patients.^21^, ^22^, ^23^ These studies have identified the relationship between lower HGB levels post‐aSAH and an elevated risk of adverse patient outcomes, establishing a well‐founded hypothesis that links reduced HGB levels to and heightened risk of cerebral hypoxia.^15^

However, limited research has focused on the influence of preoperative HGB concentration on the prognosis of aSAH patients, and the relationship between HGB concentration and clinical outcomes remains contentious. A study in 2006, examining HGB levels from day 0 (the day of SAH occurrence) to day 14 demonstrated that higher day 0 HGB levels were associated with a reduced risk of cerebral infarction, independent of vasospasm.^24^ Conversely, a 2019 study revealed that elevated preoperative blood HGB levels were linked to an increased risk of cerebral ischemia, pulmonary embolism, DVT, and unfavorable outcome among poor‐grade high‐altitude aSAH patients following clipping.^25^ Another study in 2022 indicated that patients with higher HGB levels upon admission may experience a greater incidence of DCI and DVT during hospitalization, leading to unfavorable outcomes at 90 days.^26^ It is worth noting that both studies did not classify HGB levels according to the WHO criteria (one study employed HGB = 160 g/L as the cutoff value to differentiate between standard and elevated HGB groups, while the other treated HGB as a continuous variable without grouping patients). Furthermore, both studies did not specifically delineate and analyze patients with anemia, which may limit the generalizability of their findings. Our study has contributed new evidence indicating that when we incorporate confounding factors into our analysis, abnormal HGB levels exhibit no discernible association with adverse outcomes. However, higher HGB levels are associated with an increased propensity for DVT.

Individuals with aSAH may confront an elevated susceptibility to DVT due to immobility. The bleeding subsequent to aneurysm rupture induces a prothrombotic state, thereby amplifying the risk of DVT.^27^ Notably, three studies have reported DVT incidences of 24%, 9.7%, and 3.5% in aSAH patients, respectively.^28^, ^29^, ^30^ In our study, the incidence of DVT stood at 8.6%, revealing that patients experiencing DVT during hospitalization endured prolonged stays, augmented hospital expenses, and increased in‐hospital complications. These factors compounded the medical burden on both patients' families and society at large.

Presently, there exists limited knowledge regarding the temporal patterns of DVT formation following aSAH. Familiarity with the timing aids clinicians in implementing targeted preventive measures. A 2015 study indicated that DVT formation predominantly occurs within the initial 2 weeks following aSAH, with the peak period ranging from days 5 to 9—consistent with our findings.^31^ Within our study, patients were categorized into three groups based on hemoglobin (HGB) levels, and the median duration for DVT formation in the anemic, standard, and elevated HGB groups was 7, 5.5, and 10.5 days post‐admission, respectively.

It is imperative to recognize that DVT often serves as a precursor to pulmonary embolism (PE). Among DVT patients, two individuals (2.4%) suffered from PE and succumbed. Given that PE can induce acute clinical deterioration and unexpected death, rescue interventions hold limited efficacy once emboli dislodge and trigger PE.^32^ Hence, early prevention of DVT assumes paramount importance.

Approaches to DVT prevention encompass in‐hospital health education (e.g., early lower limb muscle training), mechanical therapies (e.g., sequential compression devices), and pharmacological treatments employing anticoagulant agents (e.g., unfractionated heparin, low‐molecular‐weight heparin). However, it is vital to note that the utilization of anticoagulant agents may heighten the risk of intracranial and extracranial bleeding. Consequently, future research endeavors should aim to formulate more informed guidelines regarding the timely administration of anticoagulant agents to prevent DVT occurrence while mitigating the associated risk of hemorrhagic events.^33^

The underlying mechanism governing the formation of DVT subsequent to aSAH remains enigmatic. A study conducted in 2020 suggested that an elevated d‐dimer level upon hospitalization might augment the likelihood of DVT, implying a robust initial coagulation activation resulting in a hypercoagulable state.^34^ Furthermore, factors such as advanced age, African American ethnicity, male gender, and prolonged stays in the intensive care unit have been linked to an increased risk of DVT.^30^ Our investigation introduces novel insights into the identification of individuals predisposed to DVT, with a specific emphasis on patients presenting with elevated HGB levels upon admission, who are 2.4‐fold increased susceptibility to developing DVT during hospitalization compared to those with standard HGB levels. To date, the precise mechanism through which heightened HGB concentrations contribute to DVT formation remains elusive. One plausible hypothesis posits that elevated HGB function as a risk factor for heightened blood viscosity, thereby inducing a hypercoagulable state and facilitating thrombosis within the deep venous system of aSAH patients with elevated HGB levels, particularly during prolonged periods of immobility and reduced circulatory flow. Nevertheless, further research is warranted to validate this hypothesis and ascertain whether patients with elevated HGB can derive benefits from early anticoagulant therapy.^35^

## 5 LIMITATIONS

This study harbors several limitations. Firstly, despite adjusting for numerous confounding factors, the presence of pre‐existing medical conditions that may influence hemoglobin concentrations remains unknown. Secondly, the investigation did not delve into the specific subtypes of anemia. Thirdly, the number of patients with elevated HGB was relatively small, potentially impeding the generalizability of the results. Fourthly, data concerning daily HGB values during the initial stages of hospitalization were not collected. Fifthly, the analysis solely encompassed patients with a solitary aneurysm.

## 6 CONCLUSION

In this expansive, single‐center, observational cohort study conducted in China, 6.4% of patients exhibited elevated HGB levels upon admission, in accordance with the criteria set forth by the World Health Organization. Following adjustment for clinical confounding factors, abnormal HGB levels demonstrate no association with adverse outcomes at 90 days mark. However, it is worth noting that patients who presenting with elevated HGB upon admission exhibited an increased risk of DVT. Our findings furnish robust clinical evidence for identifying individuals at an increased risk of DVT and offer fresh insights into early prevention strategies for DVT in patients suffering from aSAH.

## AUTHOR CONTRIBUTIONS

**Conception and design:** Yuanli Zhao, Xiaolin Chen, Jizong Zhao. **Acquisition of data:** All authors. **Analysis and interpretation of data:** All authors. **Drafting the article:** Fa Lin, Changyu Lu. **Reviewed submitted version of manuscript:** Yuanli Zhao, Xiaolin Chen, Jizong Zhao. **Statistical analysis:** Fa Lin, Changyu Lu, Runting Li, Heze Han, Yu Chen. **Administrative/technical/material support:** Yuanli Zhao, Xiaolin Chen, Jizong Zhao. **Study supervision:** Yuanli Zhao, Xiaolin Chen, Jizong Zhao.

## FUNDING INFORMATION

This study was supported by the National Key Research and Development Program of China (Grant Nos. 2021YFC2501101 and 2020YFC2004701), Natural Science Foundation of China (Grant No. 82202244), and Bai Qian Wan Talent Plan (Grant No. 2017A07).

## CONFLICT OF INTEREST STATEMENT

The authors have no personal, financial, or institutional interest in any of the materials or methods used in this study or the findings specified in this paper.

## Supporting information


Table S1.

## Data Availability

The data that support the findings of this study are available from the corresponding author upon reasonable request.
