# Supplementary material for: The association between hemoglobin concentration and clinical outcomes after aneurysmal subarachnoid hemorrhage: Insights from the LongTEAM registry
Source: CNS Neurosci Ther. 2023 Oct 17;30(4):e14506. doi: 10.1111/cns.14506 (PMC11017457; doi:10.1111/cns.14506)
Supplement: Supplementary file 1 — Table S1. [file CNS-30-e14506-s001.docx]

**Supplemental Table 1.** In-hospital complications and definitions

| In-hospital complications | Definitions |
| --- | --- |
| Cardiac event | The first occurrence of non-fatal heart failure, non-fatal myocardial infarction, non-fatal arrhythmia, non-fatal cardiac dysfunction, or any cardiovascular-related mortality after treatment. |
| Delayed cerebral ischemia | New focal neurological deficit or global neurological deterioration (a drop of ≥ 2 points on the Glasgow Coma Scale [GCS]) lasting > 2 hours, after exclusion of intracranial hemorrhage, hydrocephalus, seizures, metabolic derangements, and infection, with or without radiological signs of cerebral vasospasm. |
| Intracranial infection | (1) The patient had clinical manifestations of various intracranial infections such as postoperative fever, headache, or neck stiffness. (2) The patient’s cerebrospinal fluid test showed inflammatory index changes, which met one of the following: white blood cell count > 0.01 × 10^9^ /L; cerebrospinal fluid protein > 4.50 g/L; cerebrospinal fluid glucose < 2.50 mmol/L. (3) The peripheral blood white blood cell count was > 10 × 10^9^ /L. |
| Stress ulcer bleeding | No previous history of peptic ulcer or peptic hemorrhage, and after treatment for aSAH, patients were tested positive by fecal occult blood test. |
| Urinary tract infection | Clinical symptoms of urinary tract infection or positive urine culture. |
| Hypoproteinemia | Total protein < 60g/L or albumin < 30g/L. |
| Pneumonia | Description of clinical indications or positive chest radiograph. |
| Deep vein thrombosis/ muscular calf vein thrombosis | Clinical diagnosis of muscular calf vein thrombosis/deep vein thrombosis supported by ultrasound or venography. |
| Lipid metabolism disorder | Abnormal levels of cholesterol, triglycerides, low-density lipoprotein, and high-density lipoprotein. |
